# Supplementary material for: Recurrence of Chronic Rhinosinusitis with Nasal Polyps After Surgery: Risk Factors, Predictive Models, and Treatment Approaches with a Focus on Western and Asian Differences
Source: Medicina (Kaunas). 2025 Sep 8;61(9):1620. doi: 10.3390/medicina61091620 (PMC12471848; doi:10.3390/medicina61091620)
Supplement: Supplementary file 1 [file medicina-61-01620-s001.zip › medicina-3820087-supplementary.pdf]

Search strategy:

```
(
    "Chronic rhinosinusitis"[Mesh] OR
    "chronic rhinosinusitis"[Title/Abstract] OR
    "CRS"[Title/Abstract] OR
    "chronic sinusitis"[Title/Abstract] OR
    "chronic rhinosinusitis with nasal polyps"[Title/Abstract] OR
    "chronic rhinosinusitis without nasal polyps"[Title/Abstract]
)
AND
(
    "recurrence"[Mesh Terms] OR
    "recurrence"[Title/Abstract] OR
    "recurrence rate"[Title/Abstract] OR
    "relapse"[Title/Abstract] OR
    "recurrent"[Title/Abstract] OR
    "revision surgery"[Title/Abstract]
)
AND
(
    "endoscopic sinus surgery"[Mesh Terms] OR
    "endoscopic sinus surgery"[Title/Abstract] OR
    "ESS"[Title/Abstract] OR
    "functional endoscopic sinus surgery"[Title/Abstract] OR
    "FESS"[Title/Abstract] OR
    "reboot surgery"[Title/Abstract] OR
    "sinus surgery"[Title/Abstract]
)
AND
(
    "risk factor"[Title/Abstract] OR
    "risk factors"[Title/Abstract] OR
    "prognostic"[Title/Abstract] OR
    "predictive"[Title/Abstract] OR
    "prediction"[Title/Abstract] OR
    "biomarker"[Title/Abstract] OR
    "biomarkers"[Title/Abstract] OR
    "treatment"[Title/Abstract] OR
```

"therapy"[Title/Abstract] OR
"management"[Title/Abstract] OR
"biologic"[Title/Abstract] OR
"biologics"[Title/Abstract] OR
"surgical revision"[Title/Abstract] OR
"postoperative care"[Title/Abstract] OR
"treatment strategy"[Title/Abstract] OR
"therapeutic approach"[Title/Abstract]
)
